# In-Hospital Outcomes of Hip Arthroplasty for Femoral Neck Fractures in Young Adult Patients: A Nationwide Study

**DOI:** 10.1155/aort/3328450

**Published:** 2025-02-23

**Authors:** Hembashima Gabriel Sambe, Urvish Patel

**Affiliations:** ^1^Department of Pharmacy, University of Washington, Seattle, Washington, USA; ^2^Department of Public Health, Icahn School of Medicine at Mount Sinai, New York, New York, USA

## Abstract

**Introduction:** Femoral neck fractures (FNFs) in young adults are relatively uncommon but pose significant clinical and surgical challenges. Hip arthroplasty is rarely used as a treatment option in this population but has seen rising use over the previous decade. This study seeks to compare hip arthroplasty outcomes among young adult patients in the United States admitted with FNF by evaluating hip hemiarthroplasty (HHA) and total hip arthroplasty (THA).

**Materials and Methods:** Using the National Inpatient Sample (NIS) data, adult patients less than 50 years old who underwent HHA or THA from 2016 to 2020 were analyzed. Both groups' postoperative length of stay (pLOS), total hospital charges, and prosthesis-related complications (PRCs), including mechanical loosening (ML), prosthesis dislocation (PD), and periprosthetic fracture (PPF), were analyzed and compared.

**Results:** Out of 174,776,205 hospitalizations between 2016 and 2020, 15,590 young adult patients had FNF, and 2970 patients (2.18%) underwent hip arthroplasty (1195 HHAs and 1775 THAs). After controlling for demographic, clinical and hospital characteristics, HHA was associated with a 22.4% longer pLOS compared to THA [rate ratio: 1.224, 95% CI: 1.183 to 1.266; *p* < 0.001]. Patients in the HHA group also had higher odds of PPF (aOR: 9.06, 95% CI: 4.21, 19.48; *p* < 0.001). Conversely, patients in the THA group had higher odds of PD (aOR: 6.00, 95% CI: 1.78, 20.24; *p*=0.004). There was no statistically significant difference in total hospital charges between the groups [cost ratio: 1.03, 95% CI: 0.995 to 1.075; *p*=0.092].

**Conclusion:** Among young adults with FNF undergoing hip arthroplasty, HHA is associated with a longer postoperative hospital stay and higher risk of PPF as a major early complication, while THA is associated with a higher risk of PD. Financial burden is comparable for both procedure groups. When hip arthroplasty is a preferred treatment for FNFs, individual patient factors are important considerations that should guide the choice of procedure.

## 1. Introduction

Femoral neck fractures (FNFs) in young adults < 50 years are relatively rare fractures that often result from high-velocity trauma and may be a part of poly-trauma, with multiple fractures, including the ipsilateral femur [1, 2]. FNF patterns in the elderly differ from FNF patterns in young adults. Due to poor bone quality and low-energy injury mechanisms in elderly patients, a subcapital or mid-cervical FNF pattern is more common. Young adults typically have a basi-cervical, vertically oriented FNF pattern because of better bone quality and a higher energy mechanism [3, 4].

Over 330,000 hip fractures, half of which are FNFs, occur annually in the United States (U.S.) and are projected to double by 2050 [1, 5, 6]. Only 2%-3% of all FNFs occur in young adults [3]. Of all young adults with FNFs, only about 4% are treated with hip arthroplasty [7]. However, recent study findings indicate a rising use of hip arthroplasty, especially total hip arthroplasty (THA), among young adult patients in their 40s [8].

Depending on injury type, operative options for FNF include in situ fixation, closed or open reduction and internal fixation (CRIF and ORIF), hip hemiarthroplasty (HHA), and THA [1]. Due to concerns with functional outcomes and implant longevity, HHA and THA are typically avoided as first-line operations in young patients, and the consensus favors internal fixation, which salvages the femoral head [9, 10]. However, femoral head-preserving surgeries come with complications (such as nonunion, malunion, avascular necrosis, surgical site infection, and implant failure) affecting 5%–20% of patients and necessitating revision or conversion to hip replacement [11].

For young adult patients with risk factors for poor bone quality or failed fixation, hip arthroplasty may be performed: (1) THA may be considered depending on pre-morbid mobility and cognitive status; (2) Bipolar HHA may be preferable to THA if such patients have alcohol abuse because HHA might minimize the risk of dislocation associated with alcohol withdrawal or postsurgical intoxication [12]. Recently, more literature has reported the use of hip arthroplasty as first-line operation in patients under the age of 50 [8].

Unlike most existing studies focusing on hip arthroplasty for FNF in older populations, substantial gaps exist in the current knowledge of clinical outcomes of the few young adult patients with FNF who undergo hip arthroplasty as initial surgical treatment. This dearth of information may hinder clinical decision-making and resource allocation [13]. Thus, based on the most current data available in the National Inpatient Sample (NIS), we aim to determine if there is a difference between HHA and THA for young patients FNFs with regard to postoperative LOS (pLOS), prosthesis-related complications (PRCs), and total charges incurred.

## 2. Methods

### 2.1. Study Design and Study Population

This is a Level of Evidence III retrospective observational study involving adult patients in the U.S. who underwent hip arthroplasty following a FNF between 2016 and 2020. Patients aged < 18 years or ≥ 50 years, as well as those who underwent other procedures for their FNF (e.g., internal fixation), were excluded. Patients with number of days from admission to primary procedure recorded as values < 0 were also excluded.

### 2.2. Study Outcomes and Endpoints

pLOS was defined as the number of days from the hip arthroplasty procedure to hospital discharge. pLOS was analyzed as a continuous variable and modeled to estimate the relative change in mean pLOS by procedure type.

PRCs, including mechanical loosening (ML), prosthesis dislocation (PD), periprosthetic fracture (PPF), and aggregate PRC, were defined as binary (yes/no) outcomes. The 10th Revision International Classification of Diseases, Clinical Modification (ICD-10-CM) codes were used to identify PRCs (Table A4) [14]. The aggregate PRC indicator signified the presence of at least one PRC in a patient. Adjusted odds ratios (aOR) of developing specific PRCs by procedure type were estimated.

Adjusted total charges was defined as the total financial cost (in U.S. dollars) billed for the index hospitalization. Consumer Price Indexes (CPIs) were used to adjust charges in prior years (2016–2019) for inflation and converted to 2020 U.S. dollars [15]. Adjusted total charges was analyzed as a continuous variable and modeled to estimate the relative difference in adjusted mean hospital costs by procedure type.

### 2.3. Comparison Groups and Comorbidities

ICD-10-CM codes were used to identify young adult patients admitted with FNF as a primary diagnosis (Table A1) [14]. The ICD-10, Procedure Coding System (ICD-10-PCS) codes were used to identify patients with FNFs undergoing HHA or THA as a primary procedure (Table A2) [16]. ICD-10-CM codes were also used to identify comorbidities or coexisting conditions of osteoporosis, obesity, diabetes mellitus (DM) with or without complications, hypertension (HTN), venous thromboembolism (VTE), tobacco use/nicotine dependence, alcohol abuse/dependence, and drug abuse/dependence (Table A3) [14].

### 2.4. Source and Details of Data

Data was obtained from the NIS, a deidentified administrative database in the Agency for Healthcare Research and Quality (AHRQ)-funded Healthcare Cost and Utilization Project (HCUP). The NIS is the largest openly accessible inpatient care database in the U.S., containing discharge-level data provided by 49 statewide data organizations [48 States plus the District of Columbia (D.C.)] participating in the HCUP [17].

The NIS dataset includes a stratified sample of 20% of discharges from all HCUP-participating hospitals, totaling seven million annual hospitalizations, approximating 35 million hospitalizations between 2016 and 2020, and 175 million when discharge weights are applied. This data estimates the coverage of 97% of discharges from nonfederal U.S. hospitals, encompassing 98% of the U.S. population [17].

The NIS was updated in 2015 to follow ICD-10-CM criteria. Each hospitalization is considered a distinct entry within the database, containing one principal diagnosis, a maximum of 39 secondary diagnoses, and 25 procedural diagnoses related to hospital admission. We employed discharge-level weights to aid in projecting national estimates alongside the requisite information for estimating variances [17].

### 2.5. Statistical Methods

We used IBM SPSS Statistics (Version 29) for all analyses, except for missing data imputation, which was performed using R statistical software (Version 4.3.2) [18].

The dataset met the missing completely at random (MCAR) assumptions for the variables age, race, median household income, pLOS, number of days from admission to the procedure, total charges, and primary expected Payer. However, to reduce the number of case-wise deletions during analysis and preserve statistical power, we utilized k-nearest neighbor imputation (k-NNI) to impute missing data. The k-value of 25 was determined by calculating the unweighted sample size square root and rounding to the nearest whole number [19, 20].

Frequencies and percentages for demographic, clinical, and hospital characteristics were calculated for each procedure group and tested for significant differences using independent *t*-tests, median tests and chi-square (*χ*^2^) tests. Trends of hip arthroplasty across procedure groups, as well as 5-year incidence of PRCs, were also assessed.

Weighted generalized linear models (GLM) were used to estimate the relative change in mean pLOS and adjusted mean total charges associated with each procedure type. The Poisson log-linear model was used for mean pLOS, and gamma with log link model was used for adjusted mean total charges [21]. Using multivariable logistic regression, the aOR were used to evaluate the association between procedure type and the respective PRCs. Demographic, clinical, and hospital characteristics that showed a significant association with procedure type were included in multivariable models as controlling variables.

Statistical significance for all tests utilized a two-sided approach, with values of *p* ≤ 0.05 deemed statistically significant.

### 2.6. Ethical Considerations

Neither Institutional Review Board (IRB) endorsement nor Informed consent was necessary. HCUP Data Use Agreements (DUAs) and relevant ethical oversight were in place for all researchers [17].

## 3. Results

**Figure 1:**
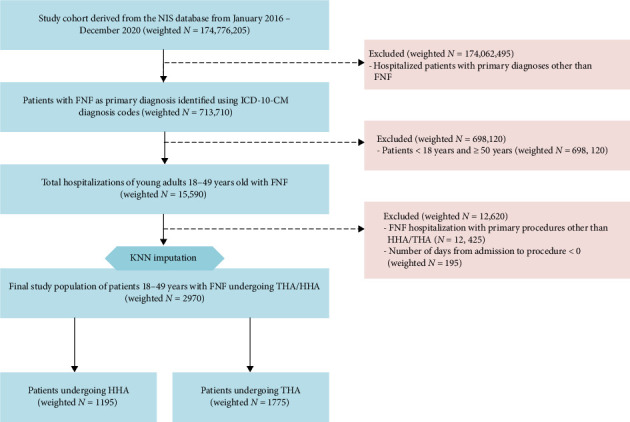
Flowchart detailing cohort selection with inclusion and exclusion criteria. ICD-10-CM = indicates international classification of diseases, 10th edition, clinical modification, FNF = femoral neck fracture; HHA = hip hemiarthroplasty, THA = total hip arthroplasty.

Among 174,776,205 patient records in the NIS from 2016 to 2020, FNF was the primary diagnosis in 15,590 young adults, representing 2.18% of all FNFs. Of these, 2970 patients (19.05%) underwent hip arthroplasty. Within the hip arthroplasty cohort, 1195 (40.2%) underwent HHA, and 1175 (59.8%) underwent THA (Figure 1).

### 3.1. Demographic, Clinical, and Hospital Characteristics

The age distributions were comparable between the HHA and THA groups, with median ages of 46 years and 45 years, respectively. Patients undergoing HHA were more likely to be covered by Medicare, while those in the THA group predominantly had private insurance. In both groups, majority of patients were White, and admissions were primarily nonelective (Table 1).

Patients undergoing THA were more likely to be obese, whereas those in the HHA group had higher rates of DM, HTN, VTE, and substance abuse or dependence (of alcohol, drugs, and tobacco). HTN and drug abuse/dependence were the most prevalent comorbidities in the HHA cohort, whereas tobacco abuse/dependence and drug abuse/dependence were the most common comorbidities in the THA group. The median number of days from hospital admission to procedure was one day in both groups but mean time to procedure was shorter for THA patients than HHA patients (Table 1).

The choice of hip arthroplasty was not affected by sex, median household income, day of admission, or osteoporosis status (Table 1).

For both procedure groups, surgery was more likely to be performed in private nonprofit, urban teaching hospitals (Table 2).

The choice of hip arthroplasty was not affected by the bed size or geographic region of the hospital (Table 2).

### 3.2. Hip Arthroplasty Trends

**Figure 2:**
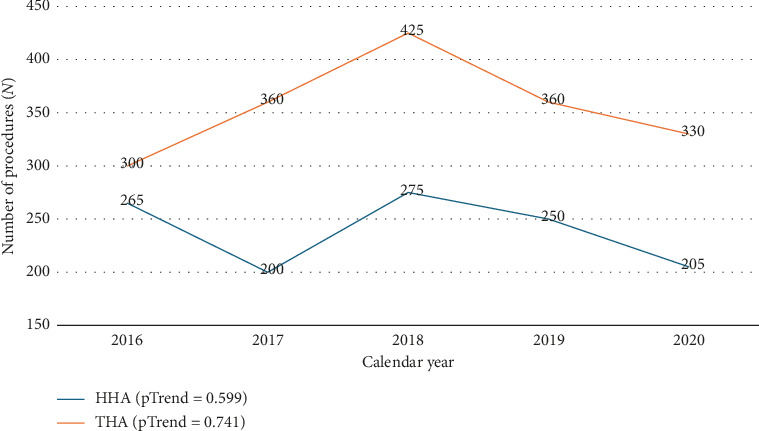
Hip arthroplasty trends for young adults from 2016 to 2020. HHA = hemiarthroplasty, pTrend = *p* value for trend in hip arthroplasty procedure over time, THA = total hip arthroplasty.

There was no significant linear trend in the number of HHA or THA procedures performed between 2016 and 2020 (Figure 2).

### 3.3. In-Hospital Outcomes by Procedure Groups

#### 3.3.1. Unadjusted Comparisons

The mean pLOS for patients undergoing HHA was 5.26 days, and the median pLOS was 4 days. Patients who underwent THA had a mean pLOS of 3.66 days and a median pLOS of 2 days (Table 3).

**Figure 3:**
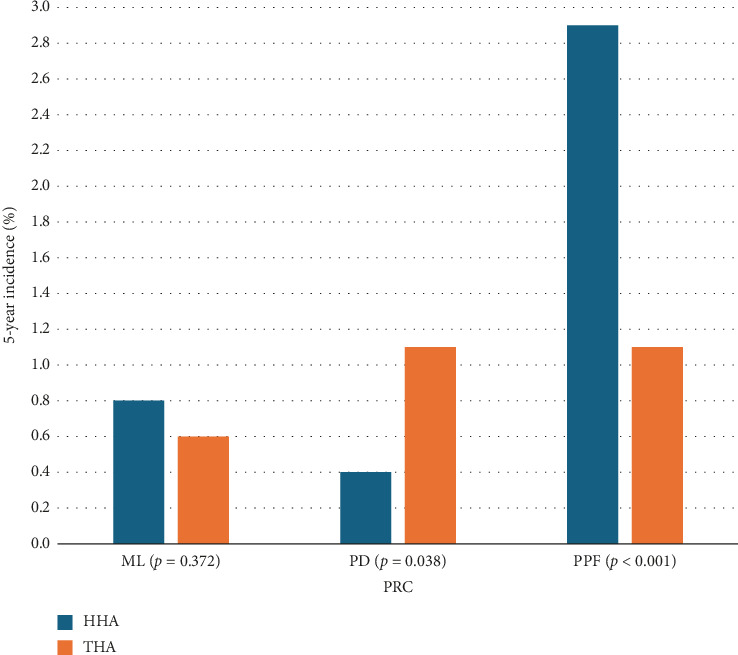
Five-year incidence of PRCs following hip arthroplasty. Statistical significance in differences indicated. HHA = hemiarthroplasty, ML = mechanical loosening, PD = prosthesis dislocation, PPF = periprosthetic fracture, PRC = prosthesis-related complication, THA = total hip arthroplasty.

PPF was the most common PRC following HHA, while PPF and PD were equally prevalent after THA. ML was the second most common PRC in patients who underwent HHA. More patients in the THA group developed PD than patients who had HHA, while patients who underwent HHA developed PPF at a higher rate than patients who had THA. There was no statistically significant difference in the 5-year incidence of ML among both groups (Table 3) (Figure 3).

Mean adjusted total charges incurred for HHA patients were lower than THA by $175.17. However, the median adjusted total charges incurred for HHA patients was higher than THA by $10,608 (Table 3).

#### 3.3.2. Adjusted Comparisons

Controlling for demographic, clinical, and hospital characteristics, multivariable GLM Poisson log-linear analysis revealed a significant relationship between procedure type and pLOS, with patients in the HHA group experiencing a 22.4% longer pLOS compared to those in the THA group (Table 4).

Multivariable logistic regression analysis adjusted for demographic, clinical and hospital characteristics, showed a significant association between procedure type and PRC for both PD and PPF. THA was associated with six times higher odds of PD than HHA while, HHA was associated with nine times higher odds of PPF compared to THA (Table 4).

No significant change in adjusted total charges between both procedure groups was observed after GLM with gamma and log link analysis, adjusted for demographic, clinical, and hospital characteristics (Table 4).

## 4. Discussion

The age range defining a young patient often spans from skeletal maturity to 50 years, though the upper limit varies among surgeons [9]. FNFs pose a significant treatment challenge in this population and while internal fixation has traditionally been the standard treatment for young patients with FNF, emerging data suggests a potential shift in these conventions [8, 13]. To our knowledge, this study is the first to utilize a national administrative database to compare in-hospital outcomes of HHA and THA among young adult patients in the U.S.

Our observation of a one-in-five rate of hip arthroplasty in young adults with FNFs represents a fivefold increase compared to prior estimates by Johnson et al., who surveyed orthopedic surgeons primarily in North America and Europe [8]. Similarly, Johnson et al. reported a fourfold increase (from 5.3% to 22.3%) in the use of THA between 2002 and 2014 [8], and Maman et al. documented a 19% THA rate among patients aged 35–44.9, a subset of our study population [13]. This rising use of THA likely reflects the durability of total hip implants as well as risks of avascular necrosis and hardware failures with internal fixation [8]. Our study uniquely identifies a higher-than-expected use of HHA, likely driven by the higher prevalence of comorbidities as well as alcohol abuse or dependence, which affected one out of every six patients in this cohort [12].

Our adjusted models demonstrated significantly higher pLOS among patients who underwent HHA compared to THA. Prior studies on pLOS for FNFs, largely focused on older populations, have shown mixed results [22–24]. Voskuji et al. observed a higher pLOS risk among hip arthroplasty patients with medical comorbidities, despite comparable pLOS between HHA and THA [24]. In our study, HHA patients had more comorbidities than THA patients, which may explain their longer pLOS.

PPF was the most common PRC overall, with patients in the HHA group having a significantly higher adjusted risk of PPF than patients in the THA group. This mirrors findings in previous literature where osteoporosis and wide femoral canals were identified as significant contributors to PPFs in patients who underwent HHA [25]. The relatively low incidence of PD in our study compared to previous studies was unexpected [26]; however, the higher adjusted odds of developing PD after undergoing THA than HHA was consistent with previous studies [26]. Surgical approach is known to influence hip stability, and the anterolateral approach is often recommended given the higher dislocation rates of the posterior approach [27].

Total charges recorded in the NIS vary from patient to patient but typically include charges for hospital rooms, supplies, medications, laboratory fees, and care staff (such as nurses). Total charges may include emergency charges prior to hospital admission but typically exclude professional fees (such as those for doctors) and noncovered expenses [17]. After adjusting for patient and hospital factors, we found no significant change in the adjusted total hospital charges between the procedure groups. In contrast, Slover et al. revealed that HHA patients, on average, had significantly lower costs than THA patients ($57,034 vs. $72,840) [28]. Other studies suggest that beyond the immediate post-op period, HHA may incur higher long-term costs than THA due to treatments for failed HHA or conversion surgeries [29, 30].

This study has several limitations. First, as a retrospective analysis of hospital admission data, it is restricted to inpatient events, making it difficult to assess the duration of FNF symptoms before presentation or long-term postsurgical outcomes. Second, the dataset lacks operative details such as anesthesia type, surgical duration, or blood loss, which are important considerations in surgical research [31]. Third, the small sample size of some PRCs resulted in a quasi-complete separation between PRCs and certain independent variables. To address this, we collapsed problematic variable levels for improved model stability [32].

## 5. Conclusion

In young adults with FNFs undergoing hip arthroplasty, HHA is associated with a longer pLOS and a higher risk of PPF, while THA is linked to a higher risk of PD. The costs between the two procedures are comparable. When hip arthroplasty is indicated, whether as a primary intervention or a revision after failed internal fixation, patient-specific factors should guide the choice of procedure to optimize outcomes. Further research is needed to clarify the benefits and risks of hip arthroplasty as its use continues to grow in this population.

## Figures and Tables

**Table 1:** Demographic and clinical characteristics by hip arthroplasty procedure.

Variables	HHA *N* = 1195 (40.2%)	THA *N* = 1775 (59.8%)	Total *N* = 2970 (100%)	*p* value
Demographic characteristics				
Age				
Mean age^†^	42.96 (6.34)	43.18 (5.98)	43.09 (6.129)	0.026
Median age⁣^∗^	46.0 (40.0–48.0)	45.0 (41.0–48.0)	45.0 (40.0–48.0)	0.586
Age groups (%)				0.015
Age group 18–29 years	75 (6.3)	70 (3.9)	145 (4.9)	
Age group 30–39 years	200 (16.7)	305 (17.2)	505 (17.0)	
Age group 40–49 years	920 (77.0)	1400 (78.9)	2320 (78.1)	
Sex (%)				0.102
Male	620 (51.9)	975 (54.9)	1595 (53.7)	
Female	575 (48.1)	800 (45.1)	1375 (46.3)	
Race (%)				< 0.001
White	895 (74.9)	1430 (80.6)	2325 (78.3)	
Black	140 (11.7)	135 (7.6)	275 (9.3)	
Hispanic	100 (8.4)	110 (6.2)	210 (7.1)	
Asian or pacific islander	15 (1.3)	40 (2.3)	55 (1.9)	
Native american	20 (1.7)	10 (0.6)	30 (1.0)	
Other	25 (2.1)	50 (2.8)	75 (2.5)	
Median household income for patient's ZIP code (%)				0.138
0–25th percentile	375 (31.4)	525 (29.6)	900 (30.3)	
26th to 50th percentile (median)	340 (28.5)	470 (26.5)	810 (27.3)	
51st to 75th percentile	305 (25.5)	470 (26.5)	775 (26.1)	
76th to 100th percentile	175 (14.6)	310 (17.5)	485 (16.3)	
Patient level and admission (clinical) characteristics				
Primary payer (%)				< 0.001
Medicare	465 (38.9)	330 (18.6)	795 (26.8)	
Medicaid	355 (29.7)	390 (22.0)	745 (25.1)	
Private insurance	240 (20.1)	725 (40.8)	965 (32.5)	
Self-pay	230 (6.3)	370 (8.2)	600 (7.4)	
No charge	80 (6.7)	165 (9.3)	245 (8.2)	
Other	45 (3.8)	145 (8.2)	190 (6.4)	
Admission type (%)				< 0.001
Nonelective	1125 (94.1)	1555 (87.6)	2680 (90.2)	
Elective	70 (5.9)	220 (12.4)	290 (9.8)	
Admission day (%)				0.486
Weekday	875 (73.2)	1320 (74.4)	2195 (73.9)	
Weekend	320 (26.8)	455 (25.6)	775 (26.1)	
Comorbidities/coexisting conditions (%)				
Osteoporosis	95 (7.9)	110 (6.2)	205 (6.9)	0.065
Obesity	90 (7.5)	210 (11.8)	300 (10.1)	< 0.001
DM	255 (21.3)	235 (13.2)	490 (16.5)	< 0.001
HTN	550 (46.0)	545 (30.7)	1095 (36.9)	< 0.001
VTE	40 (3.3)	20 (1.1)	60 (2.0)	< 0.001
Abuse/dependence of alcohol	200 (16.7)	200 (11.3)	400 (13.5)	< 0.001
Abuse/dependence of drug	510 (42.7)	625 (35.2)	1135 (38.2)	< 0.001
Tobacco use/nicotine dependence	470 (39.3)	625 (35.2)	1095 (36.9)	0.023
Days from admission to procedure				
Mean days from admission to procedure^⋄^	1.51 (1.69)	1.15 (1.20)	1.30 (1.43)	< 0.001
Median days from admission to procedure^♦^	1.0 (1.0–2.0)	1.0 (0.0–2.0)	1.0 (1.0–2.0)	< 0.001

*Note:* Percentages in brackets are column percentages. Percentages indicates direct comparison between HHA and THA amongst patients with FNF from year 2016–2020.

Abbreviations: DM = diabetes mellitus, HHA = hip hemiarthroplasty, HTN = hypertension, THA = total hip arthroplasty, VTE = venous thromboembolism.

^†^Expressed in mean ± SD years. Distribution is left-skewed (skewness value: −1.354).

⁣^∗^Expressed in median years with the interquartile range (IQR) in parentheses.

^⋄^Expressed in mean ± SD days. Distribution is right-skewed (skewness value = 3.102).

^♦^Expressed in median days with the IQR in parentheses.

**Table 2:** Hospital-level characteristics by hip arthroplasty procedure.

Variables	HHA *N* = 1195 (40.2%)	THA *N* = 1775 (59.8%)	Total *N* = 2970 (100%)	*p* value
Hospital level characteristics				
Bed size of hospital (%)				0.467
Small	205 (17.2)	335 (18.9)	540 (18.2)	
Medium	350 (29.3)	500 (28.2)	850 (28.6)	
Large	640 (53.6)	940 (53.0)	1580 (53.2)	
Hospital location/teaching status (%)				< 0.001
Rural	160 (13.4)	145 (8.2)	305 (10.3)	
Urban nonteaching	310 (25.9)	375 (21.1)	685 (23.1)	
Urban teaching	725 (60.7)	1255 (70.7)	1980 (66.7)	
Hospital region				0.122
Northeast	135 (11.3)	245 (13.8)	380 (12.8)	
Midwest	250 (20.9)	395 (22.3)	645 (21.7)	
South	555 (46.4)	770 (43.4)	1325 (44.6)	
West	255 (21.3)	365 (20.6)	620 (20.9)	
Control/ownership of hospital (%)				0.015
Government, nonfederal	180 (15.1)	205 (11.5)	385 (13.0)	
Private, nonprofit	840 (70.3)	1280 (72.1)	2120 (71.4)	
Private, invest-own	175 (14.6)	290 (16.3)	465 (15.7)	

*Note:* Percentages in brackets are column percentages. Percentages indicates direct comparison between HHA and THA amongst patients with FNF from year 2016–2020.

Abbreviations: HHA = hip hemiarthroplasty, THA = total hip arthroplasty.

**Table 3:** In-hospital outcomes by hip arthroplasty procedure.

In-hospital outcome	HHA *N* = 1195 (40.2%)	THA *N* = 1775 (59.8%)	Total *N* = 2970 (100%)	*p* value
Mean pLOS (days)^†^	5.26 (5.99)	3.66 (4.55)	4.30 (5.24)	< 0.001
Median pLOS (days)⁣^∗^	4.0 (3.0–6.0)	2.0 (2.0–4.0)	3.0 (2.0–5.0)	< 0.001
PRC				
-ML	10 (0.8%)	10 (0.6%)	20 (0.7%)	0.372
-PD	5 (0.4%)	20 (1.1%)	25 (0.8%)	0.038
-PPF	35 (2.9%)	20 (1.1%)	55 (1.9%)	< 0.001
-Aggregate PRC	35 (2.9%)	50 (2.8%)	85 (2.9%)	0.858
Mean adj. total charges ($)^⋄^	100,110.64 (72,873.48)	100,285.81 (69,404.55)	100,215.33 (70,808.63)	0.007
Median adj. total charges ($)^♦^	86,225.84 (58,904.00–114,145.27)	75,617.84 (57,296.06–116,572.98)	80,494.95 (58,374.00–114,791.00)	< 0.001

*Note:* Percentages in brackets are column percentages. Percentages indicates direct comparison between HHA and THA amongst patients with FNF from year 2016–2020.

Abbreviations: HHA = hip hemiarthroplasty, ML = mechanical loosening, PD = prosthesis dislocation, PPF = periprosthetic fracture, PRC = prosthesis-related complication, THA = total hip arthroplasty.

^†^Expressed in mean ± SD days. Distribution is right-skewed (skewness value = 6.551).

⁣^∗^Expressed in median days with the IQR in parentheses.

^⋄^Expressed in mean ± SD U.S. Dollars. Distribution is right-skewed (skewness value = 2.810).

^♦^Expressed in median U.S. Dollars with the IQR in parentheses.

**Table 4:** Multivariable estimates for in-hospital outcomes by hip arthroplasty procedure.

In-hospital outcome	HHA *N* = 1195 (40.2%)	THA *N* = 1775 (59.8%)	*p*-value
Mean pLOS [exp(B)]	1.224 (1.183, 1.266)	1.0 (REF)	< 0.001
PRC (aOR)			
- PD	1.0 (REF)	6.00 (1.78, 20.24)	0.004
- PPF	9.06 (4.21, 19.48)	1.0 (REF)	< 0.001
Mean adj. total charges [exp(B)]	1.0 (REF)	1.033 (0.995, 1.075)	0.092

Abbreviations: HHA = hip hemiarthroplasty, PD = prosthesis dislocation, PPF = periprosthetic fracture, PRC (aOR) = prosthesis-related complication, THA = total hip arthroplasty.

**Table 5:** Femoral neck fracture (FNF); ICD-10 CM codes.

Right	Left	Unspecified
*S72001A*	*S72002A*	*S72009A*
*S72001B*	*S72002B*	*S72009B*
*S72001C*	*S72002C*	*S72009C*
*S72011A*	*S72012A*	*S72019A*
*S72011B*	*S72012B*	*S72019B*
*S72011C*	*S72012C*	*S72019C*
*S72031A*	*S72032A*	*S72033A*
*S72031B*	*S72032B*	*S72033B*
*S72031C*	*S72032C*	*S72033C*
*S72034A*	*S72035A*	*S72036A*
*S72034B*	*S72035B*	*S72036B*
*S72034C*	*S72035C*	*S72036C*
*S72041A*	*S72042A*	*S72043A*
*S72041B*	*S72042B*	*S72043B*
*S72041C*	*S72042C*	*S72043C*
*S72044A*	*S72045A*	*S72046A*
*S72044B*	*S72045B*	*S72046B*
*S72044C*	*S72045C*	*S72046C*

*Note:* ICD-10-CM = 10th revision of the international classification of diseases, clinical modification.

**Table 6:** Hip hemiarthroplasty (HHA) and total hip arthroplasty (THA); ICD-10 PCS codes.

HHA		THA	
Right	Left	Right	Left
*0SRR019*	*0SRS019*	*0SR9019*	*0SRB019*

*0SRR01A*	*0SRS01A*	*0SR901A*	*0SRB01A*

*0SRR01Z*	*0SRS01Z*	*0SR901Z*	*0SRB01Z*

*0SRR039*	*0SRS039*	*0SR9029*	*0SRB029*

*0SRR03A*	*0SRS03A*	*0SR902A*	*0SRB02A*

*0SRR03Z*	*0SRS03Z*	*0SR902Z*	*0SRB02Z*

*0SRR07Z*	*0SRS07Z*	*0SR9039*	*0SRB039*
		*0SR903A*	*0SRB03A*
		*0SR903Z*	*0SRB03Z*

*0SRR0J9*	*0SRS0J9*	*0SR9049*	*0SRB049*

*0SRR0JA*	*0SRS0JA*	*0SR904A*	*0SRB04A*

*0SRR0JZ*	*0SRS0JZ*	*0SR904Z*	*0SRB04Z*

*0SRR0KZ*	*0SRS0KZ*	*0SR9069*	*0SRB069*
		*0SR906A*	*0SRB06A*
		*0SR906Z*	*0SRB06Z*

		*0SR907Z*	*0SRB07Z*

		*0SR90EZ*	*0SRB0EZ*

		*0SR90J9*	*0SRB0J9*

		*0SR90JA*	*0SRB0JA*

		*0SR90JZ*	*0SRB0JZ*

		*0SR90KZ*	*0SRB0KZ*

*Note:* ICD-10-PCS = 10th revision of the international classification of diseases, procedure coding system.

Abbreviations: HHA = hip hemiarthroplasty, THA = total hip arthroplasty.

**Table 7:** Comorbidities, ICD-10 CM codes.

Comorbidity	ICD-10 CM code
Osteoporosis	*M80, M81*
Obesity	*E660, E661, E662, E663, E668, E669*
DM with/without complications	*E08, E09, E10, E11, E13*
HTN	*I10, I11, I12, I13, I15, I16, I1A*
VTE	*I82, I26*
Abuse/dependence of alcohol	*F101, F102*
Abuse/dependence of drug	*F11, F12, F13, F14, F15, F16, F17, F18, F19*
Tobacco use/nicotine dependence	*Z720, F172, O9933*

*Note:* ICD-10-CM = 10th revision of the international classification of diseases, clinical modification.

Abbreviations: DM = diabetes mellitus, HTN = hypertension, VTE = venous thromboembolism.

**Table 8:** Prosthesis-related complications (PRCs): ICD-10 CM codes.

	Right	Left
Mechanical loosening (ML)	*T84030A*	*T84031A*
	*T84030D*	*T84031D*
	*T84030S*	*T84031S*

Dislocation of prosthesis (PD)	*T84020A*	*T84021A*
	*T84020D*	*T84021D*
	*T84020S*	*T84021S*

Periprosthetic fracture (PPF)	*M9701XA*	*M9702XA*
	*M9701XD*	*M9702XD*
	*M9701XS*	*M9702XS*

*Note:* ICD-10-CM: 10th revision of the international classification of diseases, clinical modification.

## Data Availability

The data supporting the findings of this study are available on Zenodo, a publicly available database, at https://doi.org/10.5281/zenodo.14523498.

## References

[1] Florschutz A. V., Langford J. R., Haidukewych G. J., Koval K. J. (2015). Femoral Neck Fractures: Current Management. *Journal of Orthopaedic Trauma*.

[2] Jain A., Sandhu H., Dhillon M. (2008). Femoral Neck Fractures. *Indian Journal of Orthopaedics*.

[3] Ly T. V., Swiontkowski M. F. (2008). Management of Femoral Neck Fractures in Young Adults. *Indian Journal of Orthopaedics*.

[4] Fischer H., Maleitzke T., Eder C., Ahmad S., Stöckle U., Braun K. F. (2021). Management of Proximal Femur Fractures in the Elderly: Current Concepts and Treatment Options. *European Journal of Medical Research*.

[5] National Center for Health Statistics NCHS (2010). Number of All-Listed Diagnoses for Discharges From Short-Stay Hospitals, by ICD-9-CM Code, Sex, Age, and Geographic Region: United States, 2010, National Health Discharge Survey Report. https://www.cdc.gov/nchs/data/nhds/10detaileddiagnosesprocedures/2010det10_numberalldiagnoses.pdf.

[6] Sing C. W., Lin T. C., Bartholomew S. (2023). Global Epidemiology of Hip Fractures: Secular Trends in Incidence Rate, Post-Fracture Treatment, and All-Cause Mortality. *Journal of Bone and Mineral Research*.

[7] Slobogean G. P., Sprague S. A., Scott T., McKee M., Bhandari M. (2015). Management of Young Femoral Neck Fractures: Is There a Consensus?. *Injury*.

[8] Johnson J. P., Kleiner J., Goodman A. D., Gil J. A., Daniels A. H., Hayda R. A. (2019). Treatment of Femoral Neck Fractures in Patients 45–64 Years of Age. *Injury*.

[9] Pauyo T., Drager J., Albers A., Harvey E. J. (2014). Management of Femoral Neck Fractures in the Young Patient: A Critical Analysis Review. *World Journal of Orthopedics*.

[10] Sprague S., Slobogean G. P., Scott T., Chahal M., Bhandari M., Young F. (2015). Neck Fractures: Are We Measuring Outcomes That Matter?. *Injury*.

[11] Jaya Raj J., Kow R. Y., Ganthel Annamalai K. (2021). Outcomes of Femoral Neck Fractures in Young Patients and the Factors Associated With Complications: A Multicenter Study from Malaysia. *Cureus*.

[12] Duckworth A. D., Bennet S. J., Aderinto J., Keating J. F. (2011). Fixation of Intracapsular Fractures of the Femoral Neck in Young Patients: Risk Factors for Failure. *Journal of Bone and Joint Surgery British Volume*.

[13] Maman D., Fournier L., Steinfeld Y., Berkovich Y. (2024). Etiology, Outcomes, and Complications of Total Hip Arthroplasty in Younger Patients: A Nationwide Big Data Analysis. *Journal of Clinical Medicine*.

[14] Centers for Disease Control and Prevention Cdc (2015). *International Classification of Diseases, Tenth Revision, Clinical Modification (ICD-10-CM)*.

[15] Federal Reserve Bank of Minneapolis (2024). Consumer Price Index, 1913-: Historical Data From the Era of the Modern U.S Consumer Price Index (CPI). https://www.minneapolisfed.org/about-us/monetary-policy/inflation-calculator/consumer-price-index-1913.

[16] Centers for Medicare & Medicaid Services (2015). International Classification of Diseases, Tenth Revision, Procedure Coding System (ICD-10-PCS). https://www.cms.gov/medicare/coding-billing/icd-10-codes.

[17] (2012). *Healthcare Cost and Utilization Project (HCUP), National (Nationwide) Inpatient Sample (NIS) Database Documentation*.

[18] R Core Team (2023). R: A Language and Environment for Statistical Computing. https://www.R-project.org/.

[19] (2015). *Healthcare Cost and Utilization Project (HCUP), HCUP Methods Series: Missing Data Methods for the NIS and the SID*.

[20] Dahl F. A. (2007). Convergence of Random-Nearest-Neighbour Imputation. *Computational Statistics & Data Analysis*.

[21] Ng V. K., Cribbie R. A. (2017). Using the Gamma Generalized Linear Model for Modeling Continuous, Skewed and Heteroscedastic Outcomes in Psychology. *Current Psychology*.

[22] Li X., Luo J. (2021). Hemiarthroplasty Compared to Total Hip Arthroplasty for the Treatment of Femoral Neck Fractures: A Systematic Review and Meta-Analysis. *Journal of Orthopaedic Surgery and Research*.

[23] Liao L., Zhao J. M., Su W., Ding X. F., Chen L. J., Luo S. X. (2012). A Meta-Analysis of Total Hip Arthroplasty and Hemiarthroplasty Outcomes for Displaced Femoral Neck Fractures. *Archives of Orthopaedic and Trauma Surgery*.

[24] Voskuijl T., Neuhaus V., Kinaci A., Vrahas M., Ring D. (2014). In-Hospital Outcomes after Hemiarthroplasty Versus Total Hip Arthroplasty for Isolated Femoral Neck Fractures. *The archives of bone and joint surgery*.

[25] Morris K., Davies H., Wronka K. (2015). Implant-related Complications Following Hip Hemiarthroplasty: A Comparison of Modern Cemented and Uncemented Prostheses. *European Journal of Orthopaedic Surgery and Traumatology*.

[26] Eskildsen S. M., Kamath G. V., Del Gaizo D. J. (2018). Age Matters When Comparing Hemiarthroplasty and Total Hip Arthroplasty for Femoral Neck Fractures in Medicare Patients. *HIP International*.

[27] Enocson A., Hedbeck C. J., Tidermark J., Pettersson H., Ponzer S., Lapidus L. J. (2009). Dislocation of Total Hip Replacement in Patients With Fractures of the Femoral Neck. *Acta Orthopaedica*.

[28] Slover J., Hoffman M. V., Malchau H., Tosteson A. N., Koval K. J. (2009). A Cost-Effectiveness Analysis of the Arthroplasty Options for Displaced Femoral Neck Fractures in the Active, Healthy, Elderly Population. *The Journal of Arthroplasty*.

[29] Kim S. H., Jang S. Y., Cha Y., Kim B. Y., Lee H. J., Kim G. O. (2024). Comparative Interrupted Time Series Analysis of Direct Medical Expense and Length of Stay in Elderly Patients With Femoral Neck Fractures Who Underwent Total Hip Arthroplasty and Hemiarthroplasty: A Real World Nationwide Database Study. *Clinical Orthopaedic Surgery*.

[30] Schmidt A. H., Leighton R., Parvizi J., Sems A., Berry D. J. (2009). Optimal Arthroplasty for Femoral Neck Fractures: Is Total Hip Arthroplasty the Answer?. *Journal of Orthopaedic Trauma*.

[31] Alluri R. K., Leland H., Heckmann N. (2016). Surgical Research Using National Databases. *Annals of Translational Medicine*.

[32] Lu X. (2016). *AmeriHealth Caritas Family of Companies, Correcting the Quasi-Complete Separation Issue in Logistic Regression Models*.

